# “Fish-in-Net”, a Novel Method for Cell Immobilization of *Zymomonas mobilis*

**DOI:** 10.1371/journal.pone.0079569

**Published:** 2013-11-13

**Authors:** Xuedun Niu, Zhi Wang, Yang Li, Zijian Zhao, Jiayin Liu, Li Jiang, Haoran Xu, Zhengqiang Li

**Affiliations:** 1 Key Laboratory for Molecular Enzymology and Engineering of Ministry of Education, Jilin University, Changchun, Jilin Province, P. R. China; 2 College of Life Science, Jilin University, Changchun, Jilin Province, P. R. China; 3 Key Laboratory for Vegetation Ecology, Ministry of Education, Institute of Grassland Science, Northeast Normal University, Changchun, Jilin Province, P. R. China; Instituto de Engenharia Biomédica, University of Porto, Portugal

## Abstract

**Background:**

Inorganic mesoporous materials exhibit good biocompatibility and hydrothermal stability for cell immobilization. However, it is difficult to encapsulate living cells under mild conditions, and new strategies for cell immobilization are needed. We designed a “fish-in-net” approach for encapsulation of enzymes in ordered mesoporous silica under mild conditions. The main objective of this study is to demonstrate the potential of this approach in immobilization of living cells.

**Methodology/Principal Findings:**

*Zymomonas mobilis* cells were encapsulated in mesoporous silica-based materials under mild conditions by using a “fish-in-net” approach. During the encapsulation process, polyethyleneglycol was used as an additive to improve the immobilization efficiency. After encapsulation, the pore size, morphology and other features were characterized by various methods, including scanning electron microscopy, nitrogen adsorption-desorption analysis, transmission electron microscopy, fourier transform infrared spectroscopy, and elemental analysis. Furthermore, the capacity of ethanol production by immobilized *Zymomonas mobilis* and free *Zymomonas mobilis* was compared.

**Conclusions/Significance:**

In this study, *Zymomonas mobilis* cells were successfully encapsulated in mesoporous silica-based materials under mild conditions by the “fish-in-net” approach. Encapsulated cells could perform normal metabolism and exhibited excellent reusability. The results presented here illustrate the enormous potential of the “fish-in-net” approach for immobilization of living cells.

## Introduction

The technique of cell immobilization plays an important role in modern biotechnology [1]–[4] because it can offer many advantages, such as enhanced fermentation productivity, feasibility of continuous processing, high cell stability, and low cost of recovery [5]–[8]. With the development of immobilization techniques, a variety of support materials have been tested for cell immobilization, including inorganic material [9], organic supports [10], and natural supports [11]. Compared with other supports, inorganic mesoporous material is advantageous in that it possesses large specific surface area, narrow pore distribution, and good resistance against biodegradation [12]. Its low cost, good biocompatibility and low toxicity [13]–[14] have also enabled many researchers to use it for cell immobilization. However, inorganic mesoporous material has seldom been used as the carrier to encapsulate living cells owing to the strenuous conditions required (extreme pH and high temperature and pressure) during the immobilization process [15], [16]. Therefore, use of inorganic mesoporous materials to encapsulate living cells under mild conditions is a challenge.

In our previous study, a general and facile approach was reported for the enzyme encapsulation in ordered mesoporous silica under mild conditions [17]. This method was named the “fish-in-net” approach because the enzyme (acting as “fish”) was gradually entrapped into the “net” produced by polymerization and condensation of performed silica precursors. The “fish-in-net” approach uses traditional inorganic mesoporous materials but a different synthesis system. It has many advantages such as encapsulating enzymes with a wide range of sizes, using non-denaturing solvent, and operating under mild conditions (at neutral pH and mild temperature and pressure). More importantly, the pore size of the “net” is smaller than the spatial size of “fish”; therefore, the entrapped “fish” cannot leach out from the “net.” This approach has been successfully used to immobilize several kinds of enzymes, including fumarase [17], trypsin [17], lipase [17], β-galactosidase [18], and hemoglobin [19]. However, the use of this technique for immobilization of living cells has not been previously reported.

**Figure 1:**
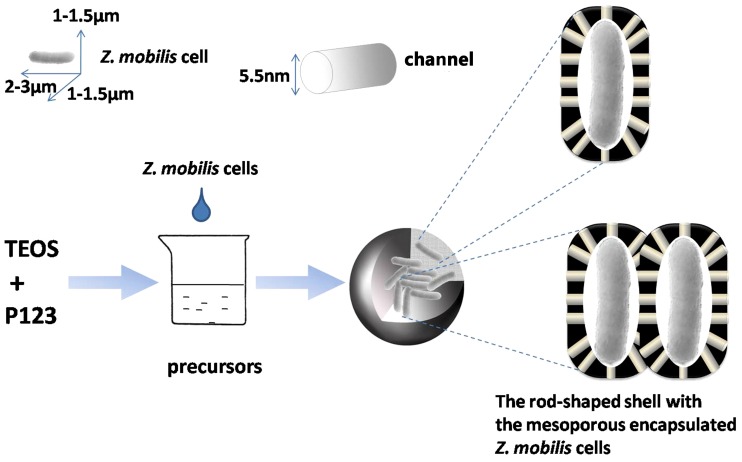
Outline of the encapsulation process by the “fish-in-net” technology.

In the present study, living cells of *Zymomonas mobilis* (*Z. mobilis*), an osmo- and ethanol-tolerant bacterium for bioethanol production [20], [21], has been selected as a model for cell immobilization by the “fish-in-net” approach under mild conditions with mesoporous silica-based materials as the carrier (Figure 1). A series of experiments have been conducted to characterize immobilized *Z. mobilis.* Furthermore, we optimized the culture conditions for immobilized *Z. mobilis* and investigated its reusability.

## Materials and Methods

### Materials

*Z. mobilis* was obtained from the China Center of Industrial Culture Collection (CICC number: 10225). Tetraethylorthosilicate (TEOS), Pluronic 123 triblock polymer [(EO)_20_(PO)_70_(EO)_20_ (Mav  =  5800)], and polyethyleneglycol (PEG 20000 Da) were commercially available from Sigma–Aldrich (St. Louis, Missouri, USA). Yeast extract was commercially available from Oxoid Company (Britain). KH_2_PO_4_, (NH_4_)_2_SO_4_, MgSO_4_·7H_2_O, HCl, ethanol, and glucose used were of analytical grade. All aqueous solutions were prepared with Milli-Q water. The glucose oxidase–peroxidase kit was purchased from the Beijing BHKT Clinical Reagent Company (Beijing, China).

### Cell cultivation

Free cells and immobilized cells of *Z. mobilis* were cultivated anaerobically for 24 h in a medium (500 ml) containing the following components: glucose (50 g), yeast extraction (2.5 g), (NH_4_)_2_SO_4_ (0.5 g), KH_2_PO_4_ (0.5 g), and MgSO_4_·7H_2_O (0.513 g).

The optimum culture conditions for free *Z. mobilis* cells were pH 5.0, 35°C, and initial glucose concentration of 100 g/L. The culture medium was adjusted to pH 5.0 with NaOH or HCl and sterilized at 110°C for 20 min. The cells were cultivated at a low mixing speed (140 rpm).

For the immobilized cells, the optimum culture conditions were pH 7.0, 30°C, and an initial glucose concentration of 100 g/l. The culture medium was adjusted to pH 7.0 with NaOH or HCl and sterilized at 110°C for 20 min. The cells were cultivated at a low mixing speed (140 rpm).

At intervals of 2 h, 5 ml of the fermentation broth of both free and immobilized *Z. mobilis* cells was collected and centrifuged (12000 rpm, 1 min) to obtain the supernatant, which was used for measuring the levels of unreacted glucose and produced ethanol.

### Immobilization of *Z. mobilis*

1) Preparation of the immobilization carrier precursor

The synthetic solution (120 ml) used was a mixture of TEOS/P123/H_2_O/ethanol/HCl/glycerol (molar ratio: 1:0.015:5.3:18.1:0.3:1.13; pH: 5.0). The mixture was fiercely stirred at room temperature until the mixture volatilized to 25% of the original volume. Then, glycerol (10 g) was added to the mixture and stirred for 1–2 h. Finally, the precursor solution was prepared.

2) Immobilization of *Z. mobilis*

*Z. mobilis* cells (0.80±0.12 g of cells, dry wt.) were harvested from the culture medium (500 ml) by centrifugation (6000 rpm, 10 min) and then washed aseptically with deionized water. Cells were resuspended in the culture medium (100 ml, containing 5% PEG, *w/v*) for 30 min. After centrifugation (6000 rpm, 10 min), the cells were transferred to a fresh culture medium (40 ml) with magnetic stirring, and this well-mixed solution was added to the precursor solution (20 ml) with simultaneous magnetic stirring at 30°C. Two hours later, the mixture was washed 5 times with the culture medium by using 5% (*v/v*) ethanol and collected by centrifugation (6000 rpm, 10 min) to remove P123. Following immobilization, the immobilized cells were repeatedly washed with distilled water to remove the un-encapsulated cells or cell debris. Consequently, immobilized *Z. mobilis* cells treated with PEG (PI-*Z. mobilis*) were obtained. Immobilized *Z. mobilis* cells that were not treated with PEG were named I-*Z. mobilis*.

### Characterization of the samples

1) Scanning electron microscopy (SEM)

Before SEM measurements, the immobilized cell samples were prepared by dispersing the powder as slurry in acetone, which was subsequently deposited and dried on a Cu plate. Free *Z. mobilis* cells were prepared in the usual manner for SEM analysis by fixation with glutaraldehyde (2%) and osmium tetroxide (1%) [22]. All the dried samples were sputter-coated with gold for SEM.

Scanning electron micrographs were obtained using a XL-30 scanning electron microscope (Philips Company, Germany) equipped with an EDAX-detector (Ametek Process and Analytical Instruments). EDAX was performed at an acceleration voltage of 20 kV.

2) Transmission electron microscopy (TEM)

Transmission electron micrographs were obtained from an FEI Tecnai G2 STwin transmission electron microscope with a field-emission gun operating at 200 kV. For TEM analysis of ultrathin sections, PI-*Z. mobilis* sample was pre-heated in a muffle furnace (450°C for 5 h) and subsequently mixed with Spurr’s resin and polymerized at 60°C for 24 h. The sample blocks were processed into 150-nm-thick sections and placed on a 200-mesh Cu grid.

3) Nitrogen adsorption/desorption analysis

After vacuum degassing at 100°C for 12 h, N_2_ adsorption/desorption isotherms of inorganic mesoporous carrier, I-*Z. mobilis* and PI-*Z. mobilis* were measured using an accelerated surface area and porosimetry system (ASAP 2020; Micromeritics, USA) at 77 K. Surface area measurements were made using the Brunauer–Emmett–Teller (BET) method in the relative pressure range (*P/P_0_*  =  0.05–0.3) [23]. Pore size distribution was calculated from the adsorption branch of the nitrogen isotherm by using the Barrett–Joyner–Halenda (BJH) method [24].

4) X-ray diffraction (XRD)

Powder XRD patterns of the samples (1 g) were recorded using a Rigaku Miniflex diffractometer with Cu Kα radiation (30 kV, 15 mA).

5) Fourier transform infrared spectroscopy (FT-IR)

The samples were mixed with KBr of spectroscopic grade and compressed into thin films. The films were approximately 10 mm in diameter and 1 mm in thickness. The FT-IR spectra was measured using VERTEX 80v vacuum FT-IR (Bruker, Germany) in the wavelength range of 400–4000 cm^–1^ for evaluation of the immobilization procedures.

6) Elemental analysis

Elemental analysis was performed on an elemental analyzer (vario MICRO CUBE; Elementar Company, Germany) by using 2 mg of the sample.

### Repeated fermentation of PI-*Z. mobilis*

At the end of each fermentation run (24 h), the recycled PI-*Z. mobilis* from the fermentation broth was washed with sterile water and transferred to a fresh medium for the next fermentation run. This procedure was continued for 10 runs.

### Measurement of ethanol and glucose

Ethanol concentration in the fermentation broth was measured by a gas chromatograph (GC-14C; SHIMADZU, Japan) with a flame ionization detector (FID). The temperatures of the detector, injector, and column were set at 240°C, 200°C, and 160°C, respectively, with a splitless time of 90 s. Nitrogen was used as the carrier gas at a flow-rate 45 ml/min. The FID consisted of a hydrogen (50 ml/min)/air (500 ml/min) diffusion flame. The injection volumes used were 1.0 µl, and propanol was used as an internal standard.

The un-reacted glucose in the fermentation broth was determined using a commercial glucose oxidase–peroxidase kit and by measuring optical density at 505 nm (UV 2550; Shimadzu, Japan).

### Calculation and statistical analysis

Immobilization efficiency was calculated using the following formula: 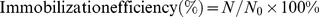



where *N* and *N_0_* reflect the nitrogen weight in the immobilized and free *Z. mobilis* cell samples, respectively.

Fermentation efficiency was calculated using the following formula:
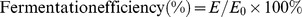



where *E* and *E_0_* reflect the actual ethanol yield and theoretical value (51.1 g/100 g glucose) [25], respectively.

All experiments were repeated 3 times, and the values in this study are given as mean ± standard deviation in triplicate for each point. One-way ANOVA was used to evaluate the reusability of PI-*Z. mobilis* with *P* < 0.05. Statistical analyses of PI-*Z. mobilis* reusability was conducted using the SAS software (SAS Institute Inc., Cary, NC, USA).

## Results and Discussion

### SEM images

**Figure 2:**
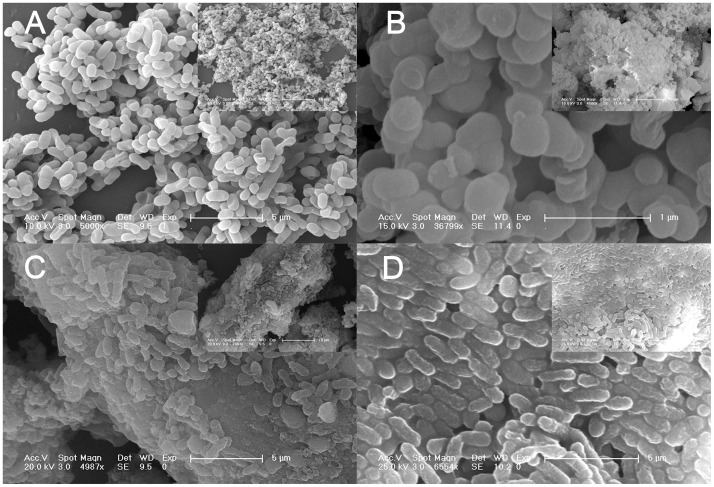
Scanning electron microscopy images. (A), (B), (C), and (D) illustrate the SEM images of free *Z. mobilis* cells, pure silica carrier, I-*Z. mobilis*, and PI-*Z. mobilis*, respectively. The insets are their respective low-magnification SEM images.

After immobilization, the microscopic morphologies of free *Z. mobilis*, pure silica carrier, I-*Z. mobilis* and PI-*Z. mobilis* were checked by SEM. The SEM image in Figure 2A clearly demonstrates that the size of a single cell was approximately 0.5×0.5 to 1.5×0.5 µm, and the cell surface appeared smooth. In Figure 2B, aggregated microspheres with a diameter of less than 500 nm were observed for the pure silica carrier. However, a rod-shaped shell was formed in the presence of living cells of *Z. mobilis*, and its size was approximately 2.0×0.5 to 2.0×1.0 µm (Figure 2C and Figure 2D). We speculated that *Z. mobilis* cells might act as a biological template to guide the precursors to form a rod-shaped shell on the cell surface.

Compared with the results shown in Figure 2C, more “rods” were formed in the presence of PEG, and its distribution was uniform (Figure 2D). The use of PEG to pretreat *Z. mobilis* cells enabled the formation of a uniform hydration layer on the cell surface. This has been shown to attract more precursors that aggregate around the cell and direct the growth of precursors to form an ordered silica shell onto the cell surface [26], [27].

### TEM images

**Figure 3:**
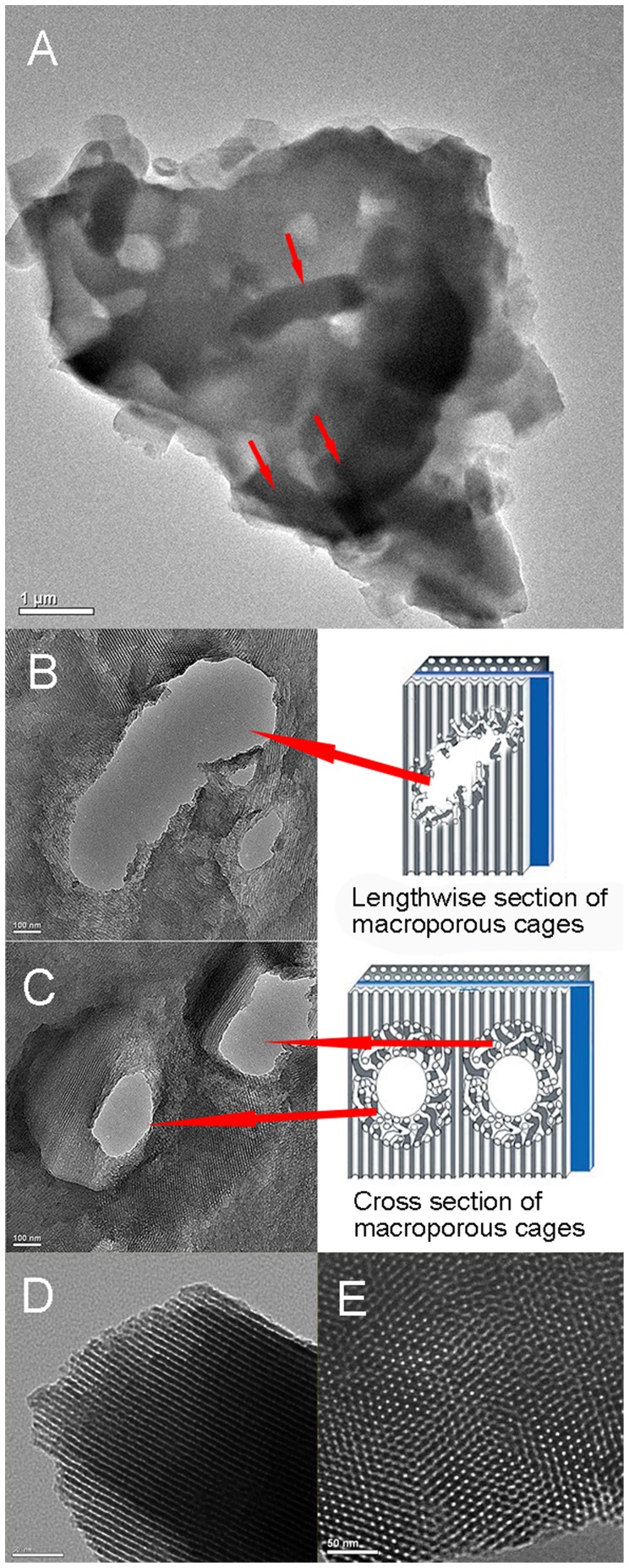
Transmission electron microscopy images. (A) shows the TEM image of PI-*Z. mobilis*. Red arrows indicate the position of *Z. mobilis* cells in the carrier. (B) and (C) are images of the ultrathin section of macroporous cages visualized by TEM from 2 different orientations [longitudinal (B) and cross-sectional views (C)]. (D) and (E) show images of the microstructure (distribution of channels) of immobilized samples checked by high-resolution TEM from 2 different orientations [longitudinal (D) and cross-sectional views (E)].

Figure 3A showed the bacilliform morphology of PI-*Z. mobilis* before calcination. In order to verify the position of *Z. mobilis* cells in the immobilized samples, the ultrathin sections were prepared after calcination and checked from different orientations by TEM (Figure 3B and Figure 3C). The macroporous cages (∼2.0×0.5 µm) can be clearly observed in the silica matrix, and its space is suitable for encapsulating a single living cell, but not for the bacteria cluster. When the bacteria cluster is encapsulated in the carrier, the cells near the surface may behave differently compared to the partially starved cells inside the carriers owing to diffusion limitation of nutrients and ethanol [28], [29]. Therefore, the single-cell encapsulation mode in this study will undoubtedly be beneficial for metabolism of *Z. mobilis* cells.

After examining the ultrathin sections, the microstructure of immobilized samples was also checked from different orientations by high-resolution TEM for in-depth understanding of the distribution of channels in the immobilized samples (longitudinal view, Figure 3D; cross-sectional view, Figure 3E). The TEM images indicated an ordered distribution of channels, which implied that the existence of living cells did not destroyed the channel structure of the silica shell.

### Nitrogen adsorption-desorption analysis

**Figure 4:**
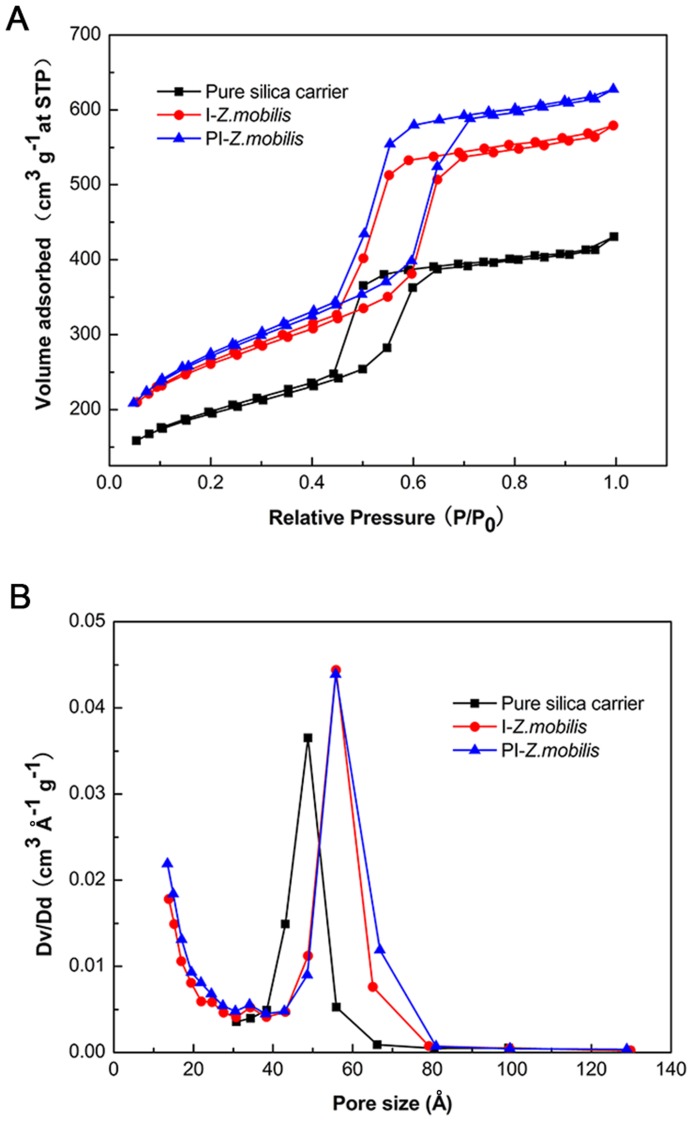
Nitrogen adsorption/desorption analysis. (A) shows the nitrogen adsorption-desorption isotherms of pure silica carrier, I-*Z. mobilis*, and PI-*Z. mobilis*, respectively. (B) indicates pore-size distribution of the pure silica carrier, I-*Z. mobilis*, and PI-*Z. mobilis* calculated from the adsorption branch of the nitrogen isotherm by using the Barrett–Joyner–Halenda (BJH) method.

The nitrogen adsorption-desorption analysis could judge the type of materials (mesoporous, microporous or macroporous) and calculate the average pore size [30]. In this study, the nitrogen adsorption–desorption isotherms and pore-size distributions of the pure silica carrier, I-*Z. mobilis*, and PI-*Z. mobilis* were investigated, and the results are presented in Figure 4. According to IUPAC nomenclature, the type-IV isotherm with a steep hysteretic loop could be observed in Figure 4A, which is typical for mesoporous materials that exhibit capillary condensation and evaporation [30], [31]. The pore-size distributions measured by the BJH method for the pure silica carrier, I-*Z. mobilis*, and PI-*Z. mobilis* were centered at 4.82 nm, 5.59 nm, and 5.63 nm, respectively (Figure 4B). These results clearly demonstrate that the pore sizes of immobilized samples were larger than those of nutrients and end products (glucose, various inorganic salts, amino acids from yeast extracts, vitamins, peptides, CO_2_, and ethanol), but smaller than those of *Z. mobilis*. Therefore, when cells were encapsulated in the inorganic mesoporous material by using the “fish-in-net” approach, they did not diffuse into the surrounding medium but could perform normal metabolism.

Compared with the pure silica carrier, I-*Z. mobilis* and PI-*Z. mobilis* show a significant increase in BET surface area and total pore volume (Table 1). It once again proved that some cells might have occupied a larger space inside the inorganic mesoporous carrier. From the data presented in Table 1, it could also be found that both BET surface area and total pore volume of PI-*Z. mobilis* are larger compared to I-*Z. mobilis*, which suggest that more cells have been encapsulated in the carrier in the presence of PEG.

**Table 1:** Structural properties of the pure silica carrier, I-*Z. mobilis*, and PI-*Z. mobilis.*

Sample	BET surface area (m^2^/g)	Pore volume (cm^3^/g)	Pore size (nm)
**Pure silica carrier**	649.9	0.6682	4.82
**I-** ***Z. mobilis***	900.2	0.8981	5.59
**PI-** ***Z. mobilis***	951.9	0.9729	5.63

### XRD analysis

**Figure 5:**
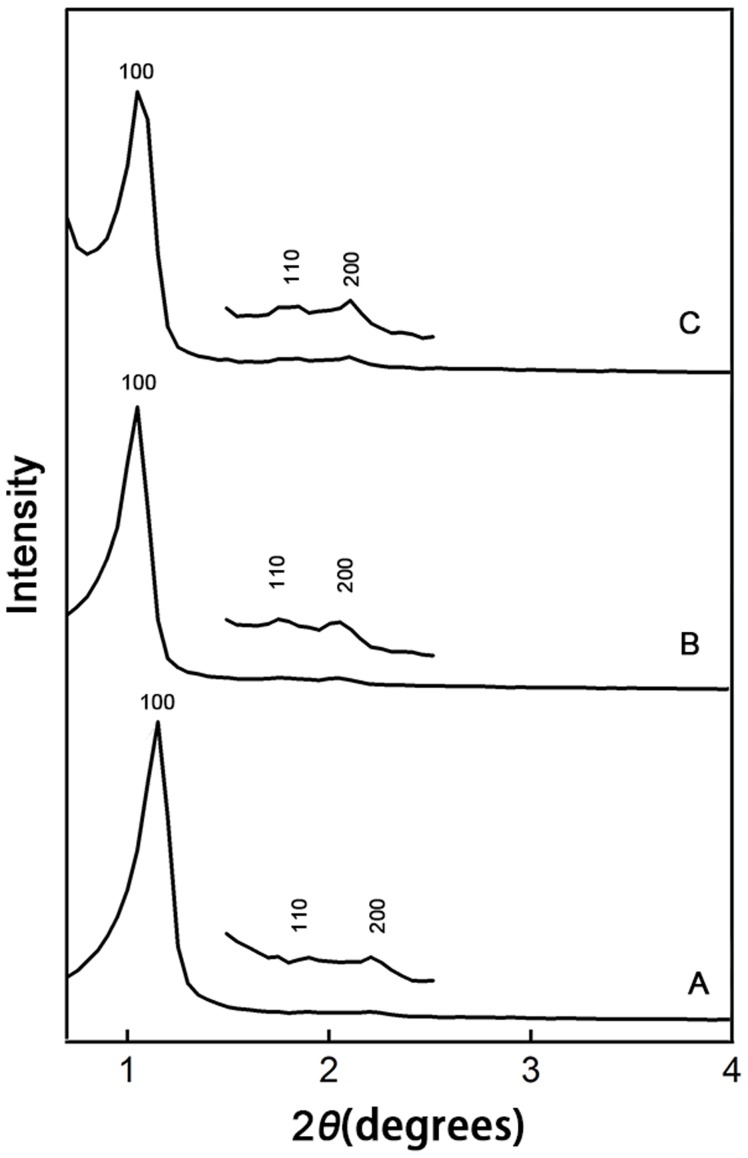
. Small-angle XRD patterns. (A), (B), and (C) show the small-angle XRD patterns of pure silica carriers, I-*Z. mobilis*, and PI-*Z. mobilis*, respectively. The weak peaks for (110) and (200) were amplified on their respective XRD patterns.

After immobilization, the structural properties of the carriers were also studied by XRD. The small-angle XRD patterns of all the samples (Figure 5) showed that three well-resolved peaks were indexable as (100), (110), and (200) reflections associated with *p6mm* hexagonal symmetry, which corresponded to the mesostructure [32], [33]. These results implied that all samples had good-order mesostructure, although the peaks (100) appeared at slightly smaller 2*θ* values, which might be due to the encapsulated *Z. mobilis* cells in the carrier.

The intense primary (100) diffraction peaks reflect the *d* spacing of 7.96 nm, 8.38 nm, and 8.48 nm for the pure silica carriers, I-*Z. mobilis*, and PI-*Z. mobili*s, respectively. The *d* spacing values were slightly higher than those obtained from nitrogen adsorption-desorption analysis. It is known that P123 is difficult to clean entirely by extraction or washing [34]. In this study, residual P123 probably resulted in reduction in the pore sizes detected by nitrogen adsorption-desorption analysis compared to those obtained from XRD.

### FT-IR spectra analysis

**Figure 6:**
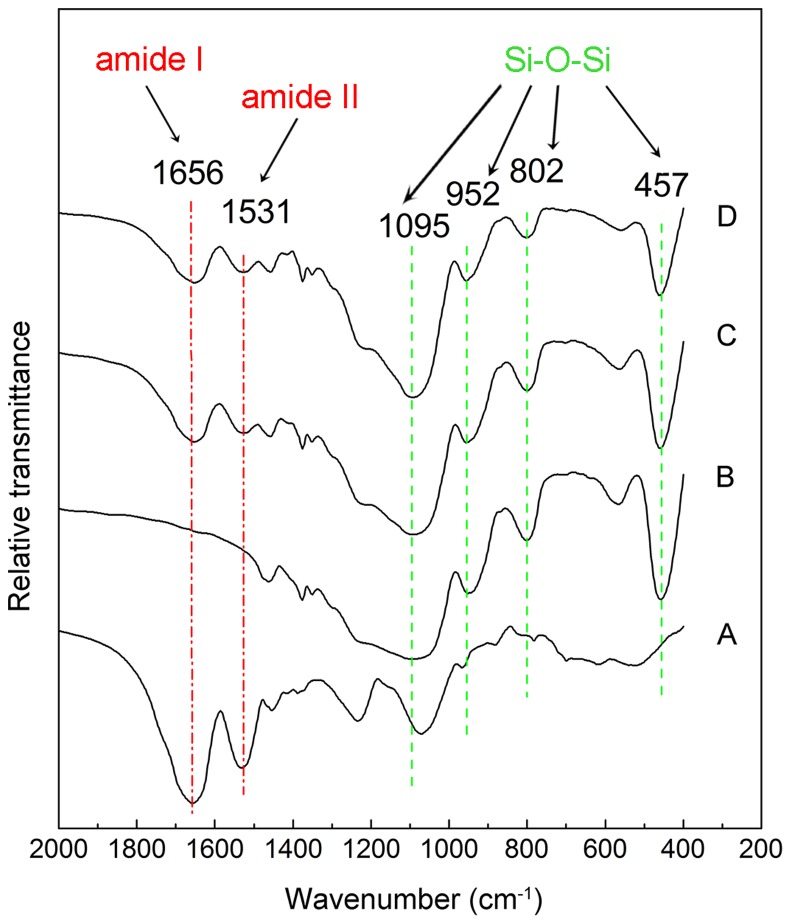
FT-IR spectra. (A), (B), (C), and (D) show the FT-IR spectra of free *Z. mobilis* cells, pure silica carrier, I-*Z. mobilis*, and PI-*Z. mobilis*, respectively. The green dashed lines indicate the characteristic peaks of Si-O-Si and the red dashed lines indicate characteristic peaks of amide I and amide II in the protein.

Infrared spectroscopy was used to confirm the presence of *Z. mobilis* cells in immobilized samples. The spectra of free *Z. mobilis*, pure silica carriers, I-*Z. mobilis,* and PI-*Z. mobilis* are shown in Figure 6.

The characteristic strong Si-O-Si stretching vibration band at 1095 cm^−1^ and three weak bands at 952, 802, and 457 cm^−1^ [35], [36] observed for pure silica carrier (Figure 6B), I-*Z. mobilis* (Figure 6C), and PI-*Z. mobilis* (Figure 6D) indicated the existence of Si-O-Si in the immobilized samples. Furthermore, the two strong absorption peaks, which were observed at 1531 (amide II band) and 1656 cm^−1^ (amide I band), associated with the secondary structure of intracellular proteins [37] in the FT-IR spectra of free *Z. mobilis* (Figure 6A), I-*Z. mobilis* (Figure 6C), and PI-*Z. mobilis* (Figure 6D) confirmed the presence of *Z. mobilis* cells in the two immobilized samples.

### SEM-EDAX

**Figure 7:**
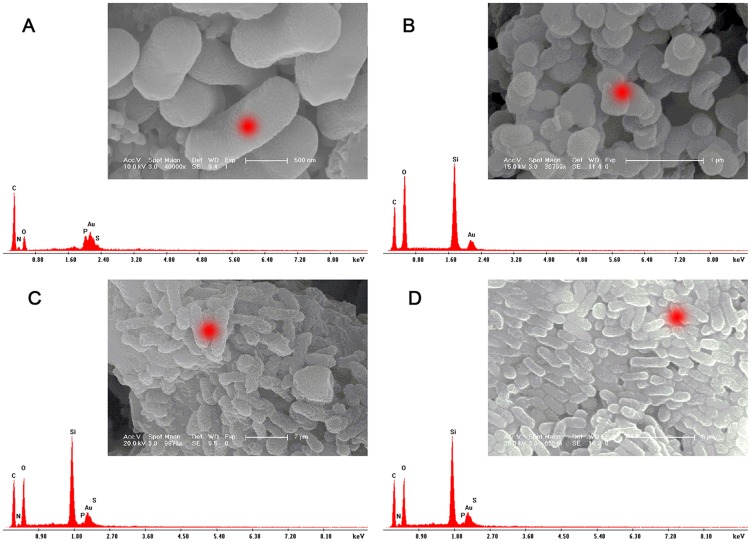
SEM–EDAX spectra. (A), (B), (C), and (D) shows the SEM–EDAX spectrum of free *Z. mobilis*, pure silica carriers, I-*Z. mobilis*, and PI-*Z. mobilis*, respectively. The red markers in their respective SEM images represent the measuring position of the X-ray.

In order to confirm that the cell of *Z. mobilis* has been completely encapsulated in the inorganic shell, SEM-EDAX was performed to determine the elemental composition of free *Z. mobilis*, pure silica carriers, I-*Z. mobilis*, and PI-*Z. mobilis* (Figure 7). In Figure 7A, carbon, nitrogen, oxygen, phosphorus, and sulfur were observed in free *Z. mobilis* cells. In Figure 7B, the presence of carbon, oxygen, and a high silicon peak confirmed the residual P123 in the pure silica carrier. In addition, carbon, nitrogen, oxygen, phosphorus, sulfur, and the high silicon peak can be observed from the red mark on the rod-shaped object in both I-*Z. mobilis* and PI-*Z. mobilis* (Figure 7C and Figure 7D). Compared with the pure silica carrier, slight changes were observed in the ratio of carbon, oxygen, and silicon for I-*Z. mobilis* and PI-*Z. mobilis*, which suggested that *Z. mobilis* cells were successfully encapsulated in the silica-based shell by “fish-in-net” approach.

### Immobilization efficiency

Results from the elemental analysis, which examined the immobilization efficiency of I-*Z. mobilis* and PI-*Z. mobilis*, are shown in Table 2. Only nitrogen could not be observed in the pure silica carrier, but it was present in *Z. mobilis*, I-*Z. mobilis*, and PI-*Z. mobilis* cells. This observation indicates that the presence of nitrogen in I-*Z. mobilis* and PI-*Z. mobilis* was because of encapsulated *Z. mobilis* cells. Furthermore, the immobilization efficiency of PI-*Z. mobilis* was 88.17%, which is 38% higher than that of I-*Z. mobilis* (63.89%); this finding implies that a higher number of cells were encapsulated in the carrier when *Z. mobilis* was pretreated with PEG. Therefore, PI-*Z. mobilis* was selected for further study.

**Table 2:** Elemental analysis for free *Z. mobilis*, pure silica carrier, I-*Z. mobilis*, and PI-*Z. mobilis*.

Sample	N [%]	C [%]	H [%]
**Free ** ***Z. mobilis***	12.65%±1.2%	45.48%±1.2%	6.64%±0.4%
**Pure silica carrier**	-	30.68%±1.2%	5.45%±0.42%
**I-** ***Z. mobilis***	0.495%±0.03%	32.1%±1.4%	5.61%±0.46%
**PI-** ***Z. mobilis***	0.69%±0.04%	32.8%±0.8%	5.63%±0.41%

### Optimization of fermentation conditions for free *Z. mobilis* and PI-*Z. mobilis*

**Figure 8:**
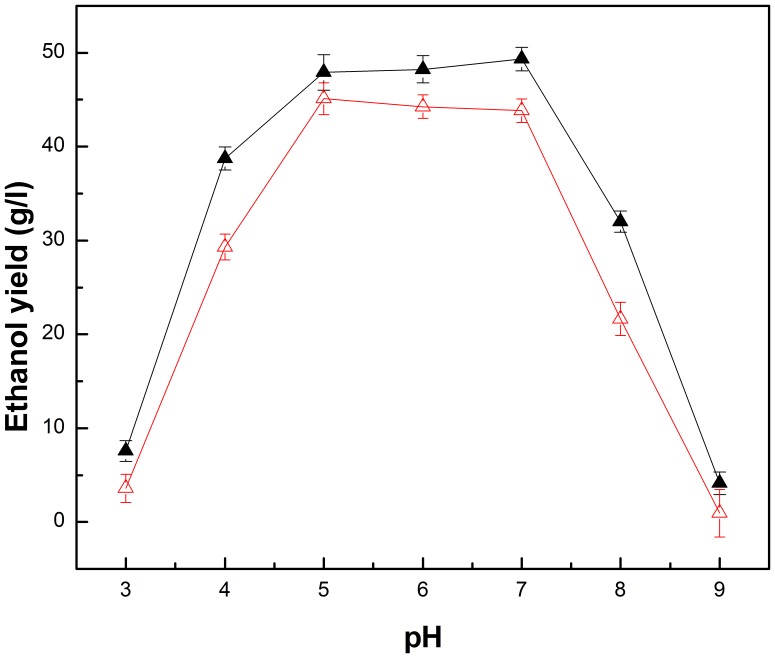
Effect of pH on ethanol yield of free *Z. mobilis* and PI-*Z. mobilis*. Fermentation of free *Z. mobilis* cells and PI-*Z. mobilis* was carried out at 35°C and at a different pH range (3–9) for 24 h, with an initial glucose concentration of 100 g/l. Ethanol yield of free *Z. mobilis* is represented as △, whereas the ethanol yield of PI-*Z. mobilis* is represented as ▴.

The pH value may change the surface electric charges of *Z. mobilis* cells and affect ethanol production of *Z. mobilis* [38]. In this study, the effect of pH was investigated in the range of 3–9 to determine the optimum pH for free *Z. mobilis* and PI-*Z. mobilis*, and the results are shown in Figure 8. We found that the optimum pH for free *Z. mobilis* was 5.0, while the optimum pH for PI-*Z. mobilis* was 7.0. The ethanol yield of PI-*Z. mobilis* increased from 7.56 g/l to a peak value of 49.33 g/l, as the pH was increased from 3 to 7. Further increasing the pH might result in a low ethanol yield.

**Figure 9:**
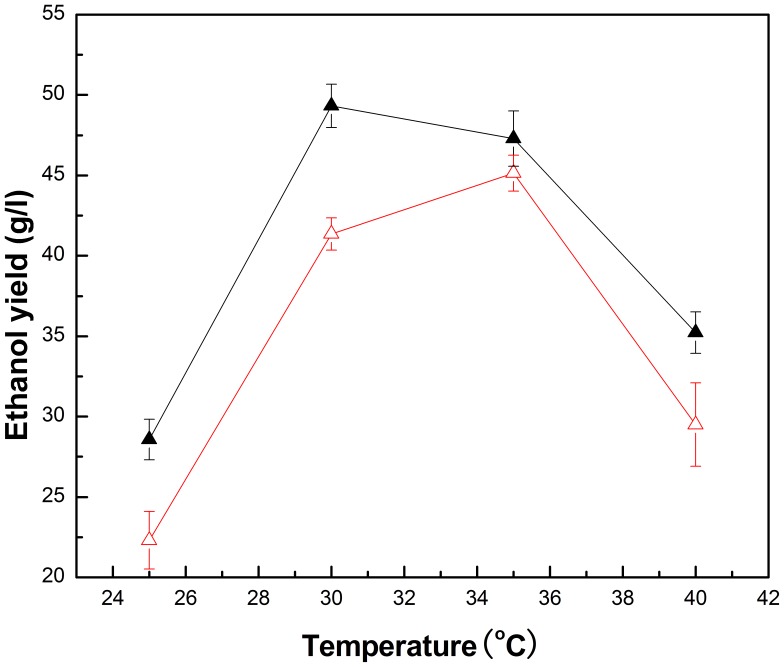
Effect of temperature on the ethanol yield of free *Z. mobilis* and PI-*Z. mobilis*. Fermentation of free *Z. mobilis* cells and PI-*Z. mobilis* was carried out at a different temperature range (25°C –40°C) at pH 5 for 24 h, with an initial glucose concentration of 100 g/l. The ethanol yield of free *Z. mobilis* is represented as △, whereas the ethanol yield of PI-*Z. mobilis* is represented as ▴.

It is well known that the ethanol yield of *Z. mobilis* is very sensitive to temperature [39]. In this study, experiments were performed in the range of 25°C ∼40°C to determine the optimum temperature for free *Z. mobilis* and PI-*Z. mobilis*, and the results are shown in Figure 9. It was found that the fermentation temperature affected the ethanol yield of free *Z. mobilis* cells and PI-*Z. mobilis* directly. The optimum temperature of free *Z mobilis* cells was confirmed as 35°C. The maximum ethanol yield of PI-*Z mobilis* (49.33 g/l) could be obtained when the temperature was controlled at 30°C. Further increasing or decreasing the temperature would decrease the ethanol yield.

**Figure 10:**
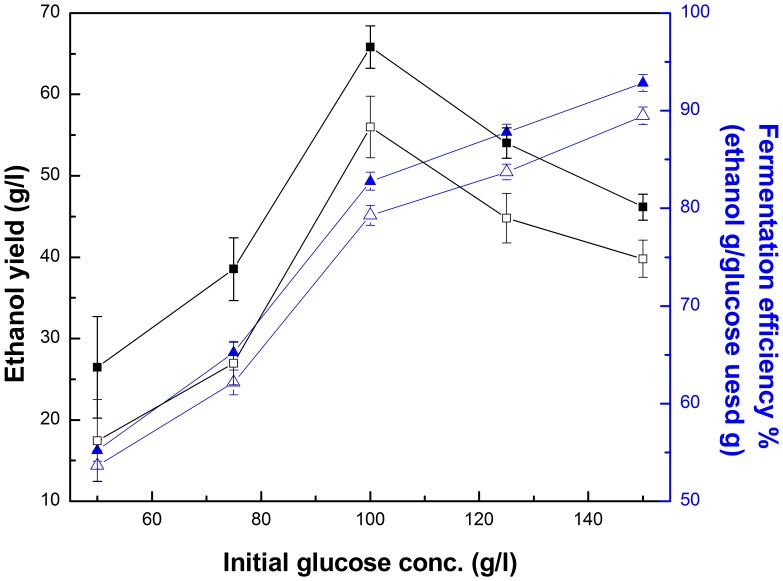
Effect of initial glucose concentration on fermentation of free *Z. mobilis* cells and PI-*Z. mobilis*. Fermentation of free *Z. mobilis* and PI-*Z. mobilis* was carried out at 35°C and pH 5 for 24 h, with a different range of initial glucose concentration (50–150 g/l). The fermentation efficiencies of free *Z. mobilis* (□) and PI-*Z. mobilis* (▪), and the ethanol yield of free *Z. mobilis* (△) and PI-*Z. mobilis* (▴) are indicated in the figure.

The effect of initial glucose concentration on the ethanol yield and fermentation efficiency was also investigated to select the optimum initial glucose concentration for the fermentation of free *Z. mobilis* and PI-*Z. mobilis* (Figure 10). Both the ethanol yield of free *Z. mobilis* and PI-*Z. mobilis* was found to increase as the initial glucose concentration was increased from 50 to 150 g/l. However, the maximum fermentation efficiency was observed at 100 g/l glucose for free *Z. mobilis* and PI-*Z. mobilis*, and it dropped at higher or lower glucose concentration. Thus, 100 g/l glucose was selected as the optimal initial glucose concentration for the fermentation of free *Z. mobilis* and PI-*Z. mobilis*.

### Comparison of the fermentation capacity of free *Z. mobilis* and PI-*Z. mobilis*

**Figure 11:**
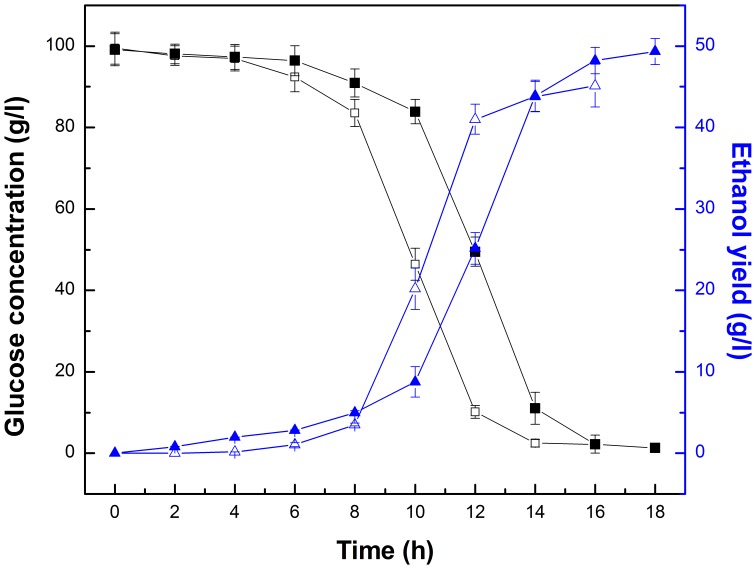
Time course of glucose consumption and ethanol production for free *Z. mobilis* and PI-*Z. mobilis*. Fermentation of free *Z. mobilis* was carried out at 35°C and pH 5, with an initial glucose concentration of 100 g/l. Fermentation of PI-*Z. mobilis* was carried out at 30°C and pH 7, with an initial glucose concentration of 100 g/l. Glucose concentration of free *Z. mobilis* cells (□) and PI-*Z. mobilis* (▪), and the ethanol concentration of free *Z. mobilis* (△) and PI-*Z. mobilis* (▴) are illustrated in the figure.

The kinetic processes of ethanol production and consumption of substrate (expressed as the reduced sugar, g/l) are shown in Figure 11. During the fermentation process, the substrates were consumed slowly within the first 4 h, which was followed by a quickly decreasing phase from 8 to 12 h and 10 to 14 h, respectively; this was consistent with the ethanol production. At the end of the fermentation period, the ethanol yield was 45.14 g/l and 49.33 g/l for free *Z. mobilis* and PI-*Z. mobilis*, respectively. The fermentation process of PI-*Z. mobilis* clearly demonstrated the presence of living *Z. mobilis* cells in the carrier.

Interestingly, the fermentation efficiency of PI-*Z. mobilis* reached 93.5%, which was 9.28% higher than that of free *Z. mobilis* cells. A possible explanation would be that immobilized cells could decrease product inhibition and then increase the fermentation efficiency.

### Reusability

**Figure 12:**
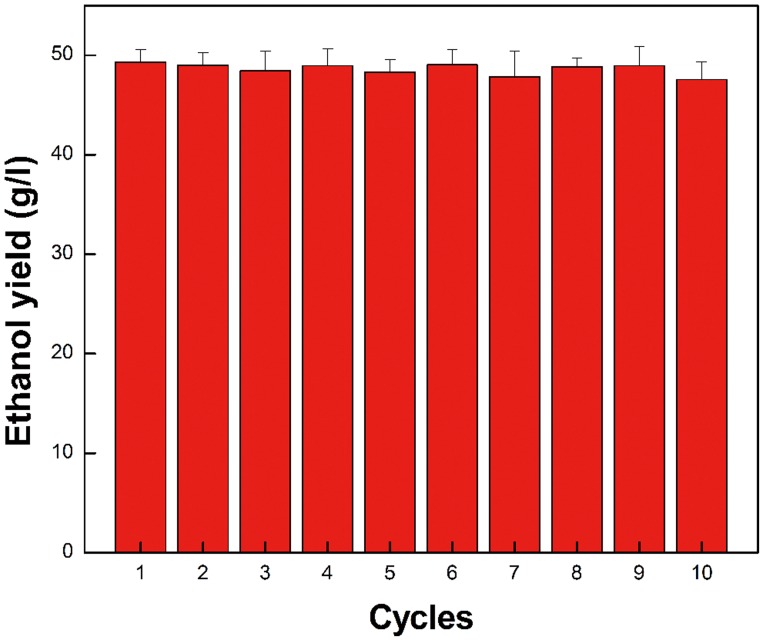
Reusability of PI-*Z. mobilis*. Fermentation of PI-*Z. mobilis* was carried out at 30°C and pH 7 for 24 h, with an initial glucose concentration of 100 g/l. At the end of each fermentation run, PI-*Z. mobilis* collected from the fermentation broth was washed with sterile water and transferred to a fresh medium for the next run of fermentation. This procedure was continued for 10 runs.

One of the most important benefits of immobilized cells is their re-use after media replacement [40]. In this study, repeated batch fermentation was performed where immobilized cells of *Z. mobilis* were recovered by centrifugation from the previous batch of fermentation media after 24 h of incubation and then transferred into a new sterile fermentation medium; this process was repeated 10 times (Fig. 12). The ethanol yield was 49.33 g/l corresponding to 93.5% of theoretical yield in run 1, whereas the ethanol yield was 46.21 g/l corresponding to 90.43% of theoretical yield in run 10. It could be concluded that there was no significant decrease of ethanol yield even after 10 runs of repeated batch fermentation (*P* < 0.05).

### Conclusions

In this study, the living cell of *Z. mobilis* has been selected as a model for cell immobilization by “fish-in-net” approach under mild conditions with mesoporous silica-based materials as the carrier. The encapsulated cells could not diffuse into the surrounding medium, but they could perform normal metabolism. Furthermore, the immobilized *Z. mobilis* exhibited excellent reusability. The results presented here illustrate the enormous potential of the “fish-in-net” approach for cell immobilization. We are currently adopting this technique to encapsulate other living cells to investigate its potential widespread utility in cell immobilization procedures.
